# Analysis of Durability of Watertight Concretes Modified with the Addition of Fly Ash

**DOI:** 10.3390/ma16175742

**Published:** 2023-08-22

**Authors:** Janina Adamus, Bogdan Langier

**Affiliations:** Department of Civil Engineering, Faculty of Civil Engineering, Czestochowa University of Technology, 69 Dabrowskiego St., 42-201 Czestochowa, Poland; janina.adamus@gmail.com

**Keywords:** watertight concrete, concrete mix, fly ash, water penetration depth, frost resistance

## Abstract

The growing demand for watertight concrete structures is conducive to the development of research in this area, but their results are rarely published. In order to partially fill this gap, the authors of the publication present the results of research into the effect of fly ash addition on the watertightness of concrete. Prior to the tests, a recipe for a concrete mix with the addition of a sealing admixture modified with fly ash was developed. The following properties were analyzed: consistency of the concrete mix, air content in the concrete mix, compressive strength of concrete, depth of penetration of water under pressure, and frost resistance of concrete for F150 level. The work meets the expectations of the construction industry with respect to the production of concrete structures resistant not only to the penetration of water into concrete but also resistant to aggressive substances dissolved in water that accelerate the destruction of concrete and corrosion of reinforcement bars. Based on the test results, it was found that the addition of fly ash to the concrete mix enhances the positive impact of the applied sealing admixture, increasing the tightness of the concrete. It reduces the depth of penetration of water under pressure and therefore increases the frost resistance of concrete.

## 1. Introduction

Although concrete is one of the oldest building materials, it is still subjected to various modifications in order to improve its physical and mechanical properties and durability in an aggressive environment. Currently, one of the basic components of concrete is modern chemical admixtures, thanks to which it is possible to optimize its properties. The variety of available chemical admixtures allows for their selection in terms of improving both the properties of the concrete mix and the durability of concrete (improving fluidity, lowering the W/C ratio without losing fluidity, sealing, aeration, accelerating or delaying setting of the concrete, increasing early strength, improving frost resistance and many other). The rapid development of chemistry also enables the use of various additives, which are materials usually used in quantities exceeding 5% of the mass of cement. They are divided into two types; one is inert additives in the form of filler of aggregates or pigments, and the other type has pozzolanic properties (silica fume, silica fly ash) or hydraulic properties (granulated blast furnace slag). The use of admixtures and additives allows modification of the concrete mix and/or hardened concrete. Concern for the environment, in accordance with the principles of sustainable development, makes it necessary to use ecological construction products that allow reducing the consumption of cement, the production of which has a negative impact on the environment, and natural construction aggregates, whose easy to extract resources, are slowly running out. In Jura et al. [1], the possibility of using fly ash from the combustion of wood–sunflower biomass as a sand substitute for the production of concrete was analyzed. In turn, Rutkowska et al. [2,3] analyzed the possibility of using fly ash from sewage sludge as an additive to ordinary concretes. Khatib et al. [4] indicate that municipal solid waste incineration bottom ash can partially substitute fine aggregate. In [5] durability of mortars with fly ash subjected to freezing and thawing cycles and sulfate attack was tested. Kelechi et al. [6] analyzed the durability performance of self-compacting concrete containing fly ash, crumb rubber, and calcium carbide waste. Raydan et al. [7] propose the use of glass powder as a material partially replacing cement. Pietrzak and Ulewicz [8] propose to use a post-consumer thermoplastic elastomer (TPE) additive derived from used car floor mats as a substitute for sand or fine aggregate. Some of the works concern special concretes in which additives play a special role, such as absorbing harmful ionizing radiation [9,10] or improving watertightness.

In recent years, the demand for building structures made of watertight concrete has been increasing rapidly. The limited availability of building lots in large cities means that many newly erected buildings have underground parking floors, which, due to the environment and harmful external conditions, need complete protection against water penetration into their structure. In addition, the intensive development of civilization causes the formation of “urban heat islands” with a much higher temperature compared to their outskirts. In order to counteract this, more and more buildings are erected with roofs covered with vegetation, which act as natural heat accumulators [11]. According to [12], green roofs can reduce the cooling load by up to 70% and provide a significant improvement in thermal comfort conditions. The environmental benefits of green roofs focus on reducing pollutant concentrations, sequestration carbon, and reducing urban noise. The positive impact of green roofs on the urban climate was demonstrated in [13].

Mousavi et al. [14] showed that the optimally designed green roof and its proper operation could result in a 12.8% increase in comfort hours and a 14% reduction in energy consumption compared to the analyzed basic variant. Additionally, by increasing the amount of green space in cities, natural habitats for bees and other pollinating insects increase [15]. The authors of the work [16] also indicate that implementing food, energy, and water production systems on urban rooftops can potentially help cities become more self-sufficient.

However, it should be remembered that the “green roof” technology requires 100% protection of the ceiling against moisture penetration. The residents of the famous Villa Savoye, designed in 1928 by the French architect Le Corbusier, learned painfully about the consequences of not meeting this condition. After a few months after its erection, they had to move out of the building, as it turned out that water leaked through the roof, and the repair process turned out to be unprofitable and difficult to carry out [17]. Certainly, the history of this building would have been different if watertight concrete and an appropriate sealing system had been used for its construction. The presented example shows that waterproof concrete should be used not only in structures made below but also above ground level when operating conditions require protection of concrete elements against the destructive effects of water. This applies to concrete structures exposed to the pressure of groundwater, such as subways, underground passages, garages, basements of buildings, water reservoirs, sewage treatment plants, as well as flat roofs or small architectural objects such as fountains.

The impact of aggressive groundwater causes corrosion and damage to concrete structures as a result of corrosion of steel reinforcement and concrete cracks. The durability of concrete structures largely depends on the resistance to water penetration and chlorine, sulfur, or carbon dioxide compounds dissolved in it. According to [18], the processes of penetration of these substances into concrete elements take place by diffusion, penetration, absorption, or electrical migration. Basheer et al. [19] reviewed the transport mechanisms, such as water absorption, permeability, and diffusion, which are responsible for the penetration of harmful substances into concrete. It has been shown that all the transport processes are interrelated and affect the strength parameters of concrete. Thus, concrete durability can be assessed based on the transport mechanisms. Transport mechanisms occurring near the surface or in the vicinity of the reinforcement play a dominant role in concrete degradation. Therefore, test methods assessing surface zone are of particular interest, especially in view of non-destructive evaluation of existing structures.

Currently, we are dealing with increasingly larger and deeper underground structures that are subject to destruction to a much greater extent than before. Unfortunately, classic concrete does not meet the waterproofing criteria. The thermal effects of cement hydration cause thermal stresses, which in turn cause the formation of a network of microscopic channels through which water easily migrates into the concrete. This is especially dangerous in the winter, when the freezing water increases in volume and bursts the concrete. Water leakage in concrete elements poses a significant issue as it shortens the structure’s lifespan and necessitates continuous maintenance. According to [20], factors contributing to water leakage through concrete structures can be divided into three groups: design, construction, and operational factors. It is usually a combination of these factors, so the problem of water leakage should be considered from the perspective of the whole system. In the case of diaphragm walls, as emphasized by Larisch [21], the method of concrete placement plays a key role.

One of the possibilities to improve the durability and reliability of concrete structures and to increase the comfort of their use is the application of modern materials and construction technologies. Until recently, the main method of extending the durability of concrete structures was to use external insulation in the form of coatings, foils, membranes, or bituminous felts [22,23,24,25] to act as a physical barrier against water. Exterior insulation tests focus primarily on the mechanical properties and chemical resistance of the materials they are made of and their adhesion to the hardened concrete surface. In recent years, in order to prevent water intrusion into new or existing concrete structures, sealants based on sodium silicate are used, which react with portlandite in the cement matrix to form calcium-silicate hydrates, partially filling the pores of the concrete. According to [26], this reaction causes an increase in the hardness of the concrete surface of the structure by about 12%. By increasing the impermeability to water by increasing the compactness of the concrete (reducing micropores, microvoids, and microcracks), the durability of the structure is extended. X. Xue et al. [27]) claim that the water-soluble hydrophobic agent they developed for impregnating concrete elements, in addition to improving water resistance, also shows excellent thermal, low-temperature, ultraviolet, alkaline, and acid resistance. It is also resistant to chlorides. Izarra et al. [28] observed that a hydrophobic release agent containing SiO_2_-CH_3_ nanoparticles dispersed in vegetable oil, used to facilitate formwork demoulding, despite the small penetration depth, improves the water resistance of concrete without affecting its compressive strength. The performance of the moisture barrier system can be significantly enhanced by the use of waterproofing/dampproofing admixtures in concrete [29]. They can effectively reduce the rate of penetration of water and aggressive chemicals dissolved in it and therefore delay the negative effects of damage caused by freezing and thawing water. For this purpose, water-reducing, air-entraining, and hydrophobic admixtures are introduced into concrete mixes. In the Muhammad et al. [30], it was found that most of the work on improving the resistance of concrete to the penetration of water and harmful substances from the environment concerns the use of polymer-based materials, silicates containing compounds, silanes, siloxanes, cementing materials, as well as nanomaterials. On this basis, the authors distinguished three groups of factors determining the watertightness of concrete, taking into account the structure of materials (macro-, micro-, and nanomaterials), methods of their application (coatings applied to dry concrete or modification of the concrete mix) and their function (thin-layer coatings reducing water absorption, membranes and pore blockers). The water absorption test is most often used to assess the impact of these factors.

At work [31], the impact of a carboxylic acid-based admixture on improving the water resistance of concrete and increasing the self-sealing capacity of the cement matrix was investigated. Li et al. [32] enhanced the anti-penetration properties of the mortar prepared from cement and powder silane water repellent thanks to the nucleation of calcium silicate hydrates (C-S-H). In turn, Geng et al. [33] propose a new method of hydrophobic treatment of concrete using SiO_2_ sol and silane emulsion. They showed that the hydrophobic effect was obtained due to the morphological changes of the concrete (C40), mainly the improvement of the concrete surface microstructure. Muwashee et al. [34] used bentonite and limestone dust as waterproofing admixtures. Among the hydrophobic agents for concrete, the most commonly used are metal soaps, wax emulsions, and liquid silane emulsions. The limitation of the large-scale use of hydrophobic additives is usually their high price and the fact that they are often damaged during use. In [35], an inexpensive and commercially available polydimethylsiloxane (PDMS) was proposed as a cement admixture for the hydrophobic modification of cement mortar. Thanks to this modification, a superhydrophobic material was obtained, which can be used in waterproof concrete, and additionally gives it anti-corrosion properties. The addition of PDMS increased the fluidity of the cement mortar, reduced the water requirement, and extended the setting time. Some researchers, including [36,37], see the solution to the problem of water permeability through concrete in the use of shrinkage-reducing admixtures and subsequent cracking of concrete, e.g., containing glycol ether, which reduces the diffusion of chlorides into concrete. Bamoharram et al. [38] showed a positive effect of nano-organic silicon compounds on improving concrete tightness. The authors of the work [39], in order to increase the water resistance of concrete, propose Dual-Crystallization Waterproofing Technology, combining hygroscopic crystallization, hydrophilic crystallization, and hydrophobic properties. They propose the use of a two-component reactive solution that can be sprayed on either fully cured or old concrete. As a result of chemical and physical reactions, water transport through the concrete matrix is minimized. Hygroscopic crystals block the pores of the concrete, reducing the space available for water.

Studies by Matar and Barhoun [40] on the impact of a waterproofing admixture on the properties of concrete made from recycled aggregate also deserve attention. Research conducted in this direction is particularly valuable due to the fact that the partial or complete replacement of natural aggregate with aggregate obtained by crushing demolished concrete is currently an innovative application in sustainable construction, as it reduces the landfill area required for waste disposal and protects natural resources by reducing the amount of aggregate extracted from pits or quarries, which was also noted by the authors of the work [41].

While watertight concretes, enhanced with specialized admixtures and additives, have been increasingly used in recent years and become impermeable to water after cur-ing, Chew and Silva [20] emphasize that an important factor determining the comprehensive watertightness of the structure is the prevention of water leakage through structural joints and expansion joints, requiring a special joint sealing and waterproofing system. The basic principles of sealing are described in [42].

Waterproof concrete structures, the so-called “White Box Concept,” are designed and built to prevent water leakage from or into the structure. B. H. Cho et al. [43] conducted a comparison of the watertightness of underground structures sealed with the traditional method of embedding PVC tapes between individual construction elements and the modified method of attaching the tape to concrete elements by means of gluing. The authors of this work emphasize that the adhesive bonding tapes provide a much better and more durable watertightness of the structure.

The literature review indicates that, at the moment, the best solution to ensure the tightness of concrete structures is the use of watertight concrete in combination with an appropriate system for sealing expansion joints and all elements included in the structure. Despite the growing demand for waterproof concretes, there are few works on their design in the available technical literature, especially in open access. Companies protect their formulas and rarely share their knowledge in this area. Therefore, the authors of this work decided to present the design of the concrete mix as well as the results of tests of the concretes intended for structures exposed to groundwater pressure, such as garages, basements, or other types of underground technical rooms. The main goal of the work is an analysis of the effect of flay ash addition on the watertightness of concrete. Our test results showed that fly ash enhanced the effect of the sealing admixture, additionally limiting the depth of penetration of water under pressure, which is extremely important not only in the case of concrete used for underground structures but also for the construction of green roofs, which is a kind of novelty on the market. In the absence of detailed guidelines for the design of watertight concretes the test results can be used by technologists and concrete designers as guidelines in the selection of the composition of watertight concrete. Experiments allow for reducing mistakes in the future.

## 2. Materials and Methods

In order to study the effect of flay ash on the watertightness of concrete., the concrete mixes with the addition of sealing admixture, which was modified with fly ash, were developed. As a part of the work, the following 4 variants of concrete mixes for making watertight concrete were designed:

- V1: reference concrete mix with an admixture of plasticizer and superplasticizer,
- V2: concrete mix of variant 1 modified with the addition of a sealing admixture,
- V3: concrete mix of variant 1 modified with the addition of fly ash used as a replacement for a part of the aggregate,
- V4: concrete mix of variant 3 modified with the addition of a sealing admixture.

The adopted designations of the mixes correspond to the order of design and execution of the tested series of concretes. First, a reference series (V1) was designed experimentally, and then, the reference series was modified with the addition of a sealing admixture and marked as V2. In the next stage, the reference series was redesigned by introducing fly ash (V3), and then a sealing admixture was added to the V3 series, and thus the V4 series was created. When designing concrete mixes, it was taken into account that the technology of water-tight concrete is effective only when we are able to eliminate possible cracks that arise not only as a result of construction errors but also improper technology of concrete production. Therefore, special attention was paid to the selection of concrete mix components, especially the selection of admixtures and additives to improve the plasticity and tightness of concrete and to ensure the proper ratio of water to cement and the optimal distribution of aggregate grains.

Experimental studies of concrete mixes and hardened concrete included testing:

- consistency of the concrete mix,
- air content in the concrete mix,
- concrete compressive strength,
- depth of penetration of water under pressure,
- frost resistance of concrete for level F150.

### 2.1. Material Properties

Portland slag cement CEM II/B-S 42.5N NA [44] was used to produce concrete, whose composition, mechanical and physical properties are presented in Table 1 and Table 2, and natural aggregate: fine with a grain size of 0–2 mm and coarse (gravel) with a grain size of 2–8 mm and 8–16 mm.

BV3M plasticizer, ViscoCrete 5 superplasticizer, and WT-200P sealing admixture were used as concrete admixtures. Silica fly ash with pozzolanic characteristics meeting the requirements of the standard PN-EN 450-1:2012 [45], i.e., loss of ignition of A category, fineness of category N, was additive to concretes. The chemical composition of fly ash is given in Table 3.

### 2.2. Concrete Mix Design

When developing the recipe for concrete mixes, the following parameters were sought: water penetration depth: <30 mm, degree of frost resistance: minimum F150, consistency class after 5 min from the first contact of water with cement: S2, air content in the concrete mix after 5 min from the first contact of water with cement: 2 ÷ 3%.

**Figure 1:**
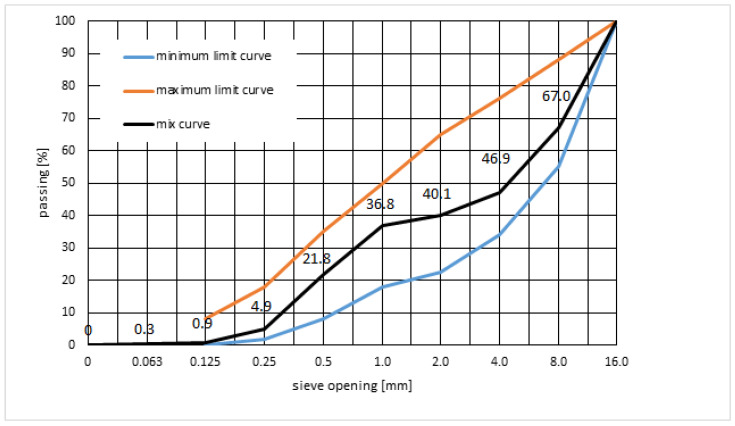
Grading curve of aggregate mix 0–16 mm with limit curves.

As part of the design of the composition of the concrete mix of variant V1, gradation tests were carried out, and then the mixing proportions were experimentally determined in order to obtain the optimal graining of the aggregate by examining its bulk density and voids. The grading curve of the aggregate mix, together with the limit curves according to PN-B-06265:2022-08 [46], is shown in Figure 1.

A sealing admixture in the amount of 1% of the cement mass was introduced into the designed composition of the concrete mix of variant V1 (variant V2). In variant V3 of the concrete mix, silica fly ash was introduced into the composition as a substitute for part of the aggregate without using a sealing admixture. As part of further modifications in variant V4, fly ash and a sealing admixture in the amount of 1% of the cement mass were used at the same time. The recipes of the tested concrete mixes are presented in Table 4.

Fly ash in V3 and V4 concrete mixes was introduced into the composition as a substitute for part of the aggregate. All designed concrete mixes meet the requirements of concrete durability in a chemically aggressive environment.

### 2.3. Methods of Testing

In order to check whether the designed concrete mixes meet the design assumptions, qualifying the concrete for use in the construction of facilities exposed to groundwater, standard tests of the obtained concretes carried out, such as consistency of the concrete mix, air content in the concrete mix, concrete compressive strength and depth of water penetration under pressure. Due to the fact that concretes are also used in cold regions exposed to excessive freezing and thawing during the year, an additional frost resistance test of concrete was carried out. The purpose of such a test is to check the resistance of concrete to the destructive effects of changing temperatures. All tests were carried out in accordance with applicable standards.

The test of the consistency of the concrete mix was carried out using the concrete slump test in accordance with the standard PN-EN 12350-2:2019-07 [47]. The consistency test of the concrete mix was carried out individually for each batch of concrete mix in the series. The assessment of the change in consistency over time was performed at the same time intervals from the moment of combining the cement with water. The concrete mix was stored in a sealed container and mixed again before testing. An evaluation of the workability of freshly made concrete up to 60 min was carried out, examining their consistency after 5, 15, 30, 45, and 60 min.

In addition to affecting the strength of concrete, the air content can also affect the migration of harmful compounds and the durability of concrete in various environmental conditions. It can also impact the finish and cosmetic appearance of concrete. Therefore, air content testing is required for concretes used for structures that may be exposed to the aggressive effects of the surrounding environment.

**Figure 2:**
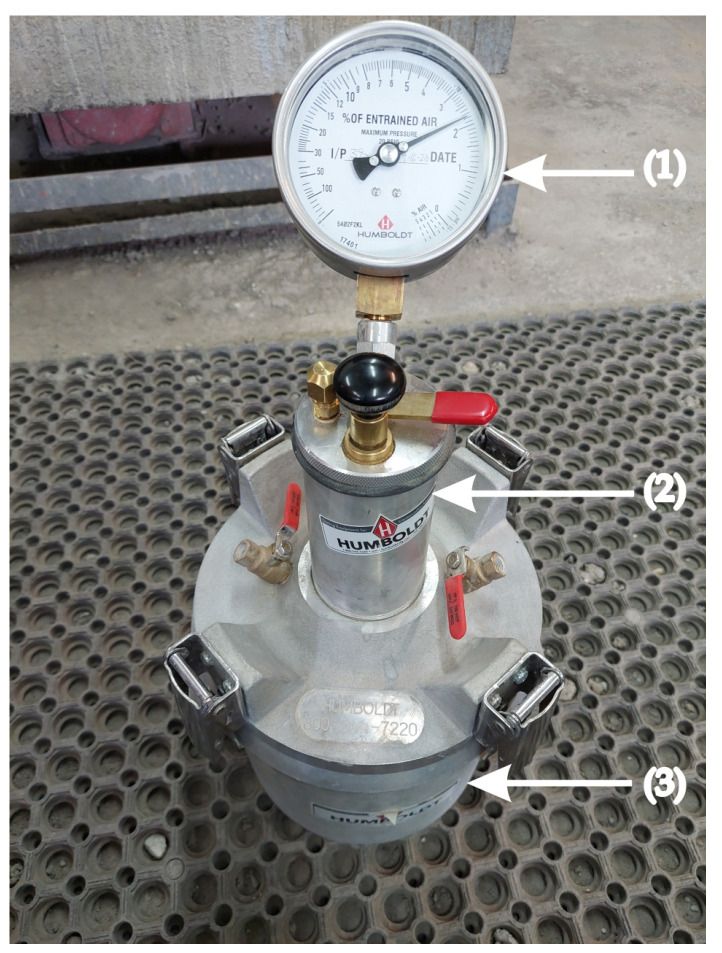
View of the device for testing air content in fresh concrete (Air Content Meter): (1) pressure gauge with aeration value indicator, (2) air chamber, (3) test container with a volume of 8 dm^3^.

The air content in the fresh concrete was tested using the pressure method (Figure 2.) in accordance with the standard PN-EN 12350-7:2019-08 [48]. The air content in individual concrete mixes was measured 5 min after the first contact of cement with water during the mixing process.

**Figure 3:**
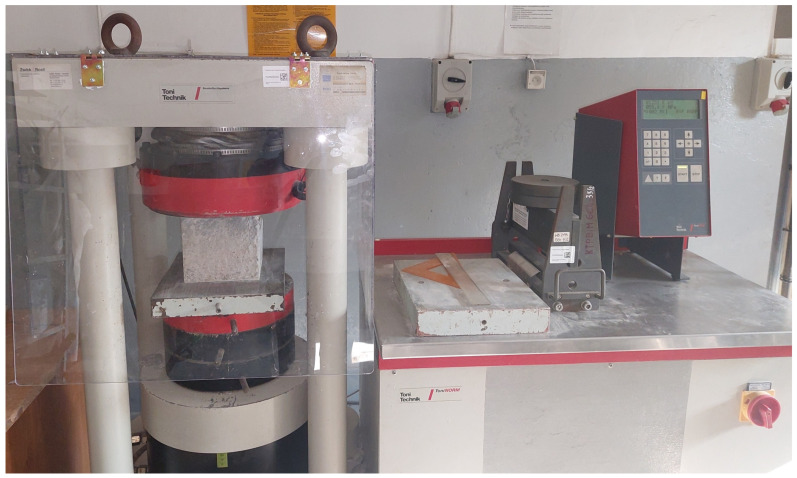
View of stand for compressive strength testing.

The compressive strength was tested on a hydraulic strength press (Figure 3) in accordance with the standard PN-EN 12390-3:2019-07 [49] using 5 cubic samples with dimensions of 150 × 150 × 150 mm and 5 cubic samples with dimensions of 100 × 100 × 100 mm, for each variant of the tested concrete.

The concrete mix was placed in molds in two layers, each of which was compacted mechanically on a vibrating table. The samples remained in the forms for 24 h. After demoulding, they were placed in water, where they were cured at the temperature of 20 ± 2 °C. In the case of cubic samples with an edge of 100 mm, the compressive strength was tested after 2, 7, 28, 56, and 90 days, and in the case of the samples with an edge of 150 mm, the tests were carried out after 7, 28 and 90 days.

**Figure 4:**
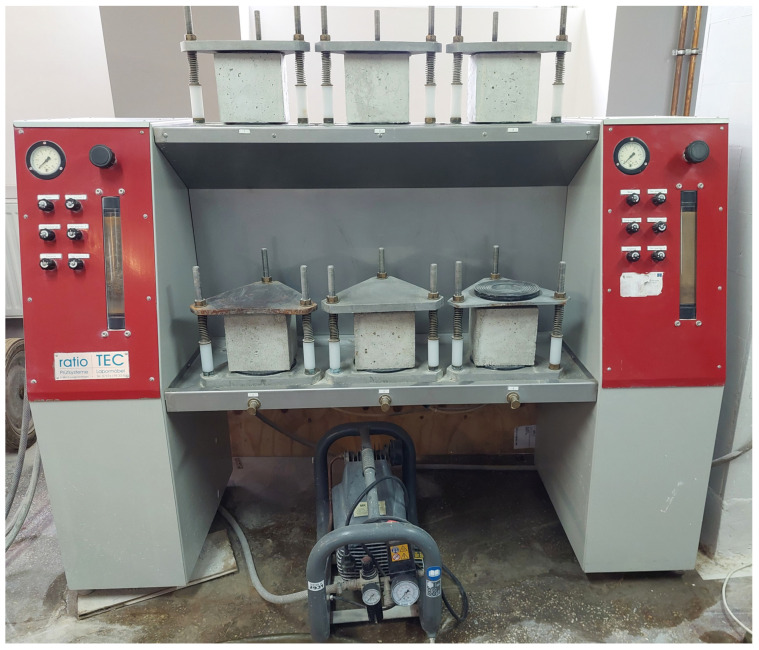
View of samples during water permeability test.

The depth of penetration of water under pressure into concrete specimens was tested in accordance with the standard PN-EN 12390-8:2019-08 [50]. The tests were carried out on cubic concrete samples with an edge of 150 mm. The tests were started after 56 days of curing concrete samples in water at a temperature of 20 ± 2 °C. Prior to testing, the samples were dried to constant weight. From the surface of the samples, where the water was pressed against the sample, cement laitance was removed from the area with a diameter of 75 mm. Then the samples were subjected to a stream of water at a pressure of 500 ± 50 kPa. A view of the samples during the water penetration test is shown in Figure 4.

**Figure 5:**
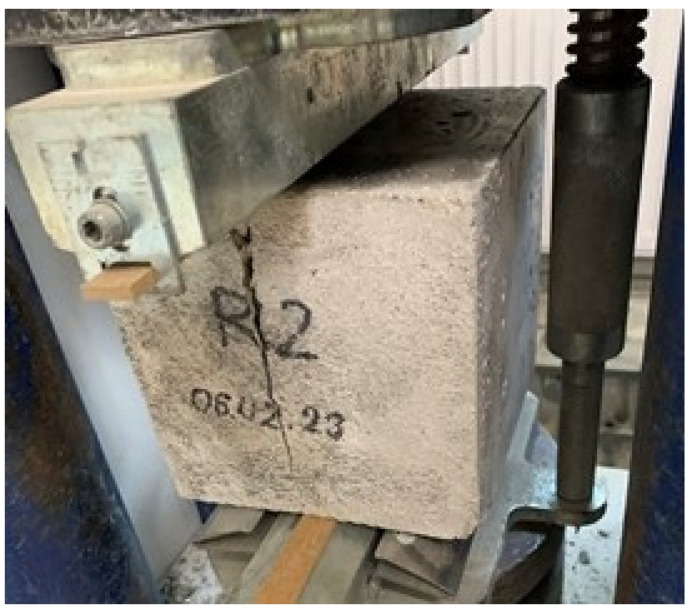
Splitting a sample after a water penetration test.

The study was conducted continuously for 72 ± 2 h. After this time, the samples were broken (Figure 5), and the depth of penetration of water was measured.

In countries such as Poland, where subzero temperatures are common in winter, it is necessary to test concrete for frost resistance because water in concrete pores freezes under the influence of negative temperatures, increasing its volume by about 10%. This causes significant stresses, leading to cracking of the concrete and loss of its tightness. Cyclic repetition of this phenomenon can lead to the complete destruction of the concrete structure. Therefore, freeze-thaw tests were carried out in accordance with the standard PN-B-06265. The tests were carried out on cubic samples with an edge of 150 mm after 56 days. The samples were subjected to 150 cycles of freezing in the air to a temperature of −18 ± 2 °C within 4 h and thawing in water at a temperature of 18 ± 2 °C for the next 4 h. After the last cycle the samples were weighed and tested for compressive strength. According to the PN-B-06265 standard, the frost-resistant level of mature concrete is achieved when, after the cycles of freezing and thawing: (1) none of the tested concrete samples shows cracks, (2) the average weight loss of the tested samples does not exceed 5%, (3) compressive strength of samples subjected to freeze-thaw cycles is not lower than 20% compared to the control samples.

## 3. Test Results and Discussion

### 3.1. Consistency of Concrete Mix

**Figure 6:**
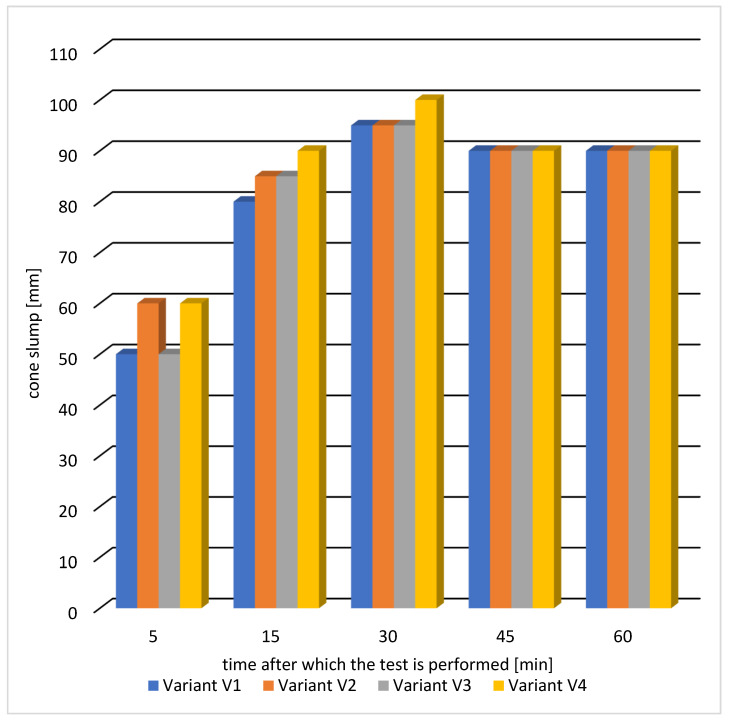
Results of consistency of the concrete mix.

The results of the consistency test are presented in Table 5 and graphically in Figure 6.

The results of testing the consistency of concrete mixes indicate that for all variants of concrete mixes, the assumed consistency class S2 was obtained after 5 min. An increased drop of the cone of the tested mixes in the time interval of up to 30 min can be seen for all tested variants. The effective action of the fluidizing admixtures was obtained after 30 min from the moment of combining the cement with water in the mixing process. A slight decrease in consistency after 30 min should be explained by of hydration of the cement binder. The concrete mix, according to variant V4, with the addition of fly ash and sealing admixture, showed the greatest liquefaction. Between 30 and 60 min, all concrete mixes retained their workability, and the cone slump was 90 mm.

### 3.2. Air Content in the Concrete Mix

The results of air content in the fresh concrete are presented in Table 6.

All concrete mix recipes met the assumption of air content within the limit of 2 ÷ 3%. Concrete mixes containing silica fly ash (variants V3 and V4) are characterized by a slightly higher air content. It can be seen that the addition of a sealing admixture reduces the air content in the tested mixes, both in the case of its introduction to the reference concrete mix (variant V1) and after its introduction to the concrete mix of variant V3 (with the addition of fly ash). In the concrete mix of the V1 variant, compared to the V2 variant, the air content decreased from 2.3% to 2.2%, whereas in the concrete mix of the V3 variant, compared to V4, there was a decrease from 2.6% to 2.4%.

### 3.3. Compressive Strength Test of Concrete

The test results of compressive strength are presented in Table 7.

The tests showed that after 28 days of concrete curing, the compressive strength class C50/60 was obtained for all four recipes, and the strengths were similar to each other. The largest difference occurred between variants V2 and V4 and amounted to about 4%. The concrete of variant V4, with the addition of fly ash and sealing admixture, obtained the highest value of compressive strength, which was 71 MPa. This may be due to the synergistic effect of the fly ash with the sealing admixture, which has an impact on the elimination of micro-cracks in concrete.

The addition of fly ash reduced the compressive strength in the first days of concrete maturation (variants V3 and V4), but in the later period, concretes with the addition of fly ash achieve a greater increase in strength, specifically the concrete according to variant V4 obtained the highest compressive strength after 90 days of maturation.

**Figure 7:**
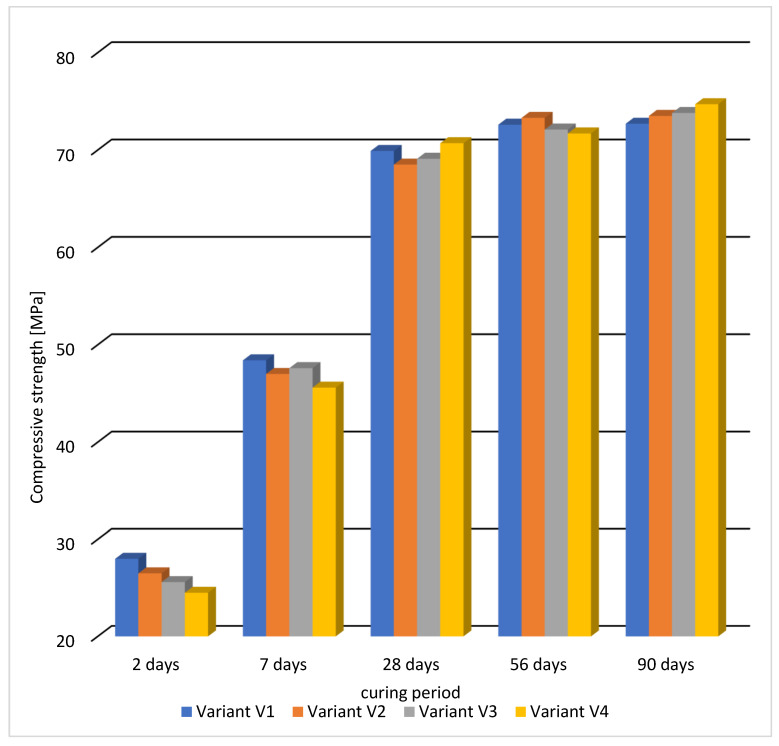
Compressive strength of concrete at various ages for samples with a side of 100 mm.

**Figure 8:**
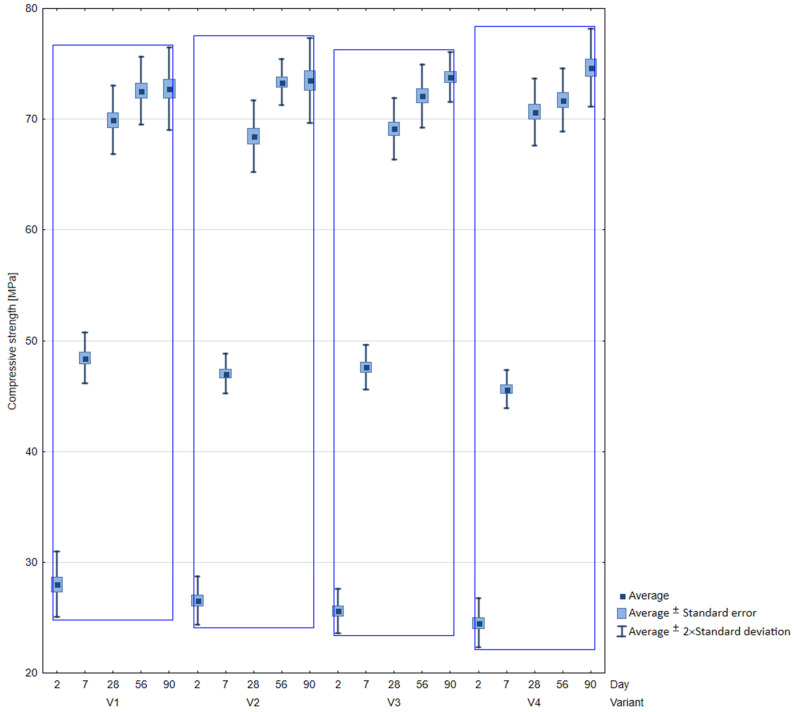
Box-plot for compressive strength results.

Figure 7 shows the change in compressive strength during the maturation of the tested variants from the 2nd to the 90th day of maturation of the cubic samples with a side of 100 mm. While Figure 8 graphically demonstrates statistical data on the results of the static compression test in the form of a box plot.

After 2 days of concrete curing, the highest strength of 28 MPa was obtained in the reference series, while in the variants with the addition of fly ash, the strengths were the lowest (variants V3 and V4). Between the 2nd and 7th day of maturation, concretes with the addition of fly ash achieved a higher increase in compressive strength compared to the series without ash, but the strengths were still slightly lower. After 28 days, very similar results of compressive strength were obtained in all variants, and the greatest increase in strength was obtained for the concrete variant V4. In the further period of concrete curing between 56 and 90 days, a further increase in compressive strength was observed for variants V3 and V4 containing fly ash.

### 3.4. Water Penetration Test

**Figure 9:**
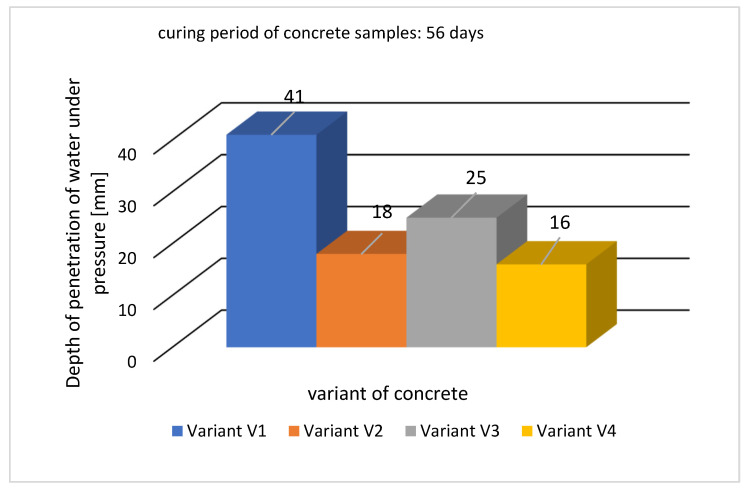
Depth of penetration of water under pressure for analyzed concretes.

**Figure 10:**
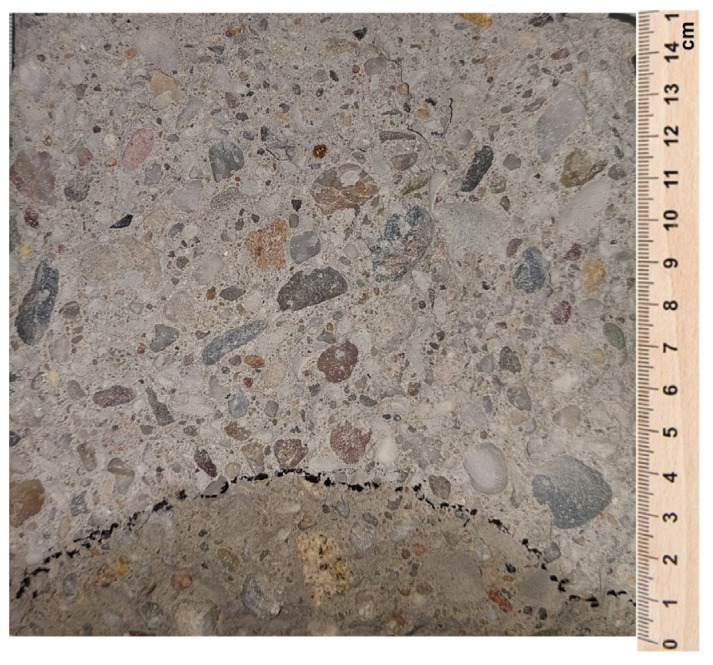
View of the concrete sample after the depth of penetration test.

The results of water penetration, i.e., the depth at which water penetrated the concrete sample, are shown in Figure 9, while an example of the appearance of the sample after fracture is shown in Figure 10.

The depth of water penetration into the concrete of the reference series (V1) was 41 mm. For the remaining modified concrete mix recipes (variants V2 ÷ V3), the assumed water tightness of concrete was obtained, i.e., the depth of penetration of water under pressure did not exceed the assumed limit of 30 mm. The analysis shows that the sealing admixture (variant V2) and fly ash (variant V3) affect the reduction in the penetration depth. Modification of the reference series of concrete (variant V1) by introducing fly ash into the concrete mix (variant V3) resulted in a reduction in water penetration by 39%, while modification of variant V1 with sealing admixture (variant V2) resulted in the reduction in water penetration by 56%. In the case of variant V4, the addition of fly ash enhanced the effect of sealing admixture, reducing water penetration by 61%. The synergy of both components effectively seals the concrete structure, creating a barrier that is hardly permeable to water.

Such concrete reduces not only the water penetration into the concrete but also prevents the ingress of aggressive substances dissolved in water, which in turn decreases the destruction of concrete and corrosion of reinforcing bars.

### 3.5. Freeze-Thaw Test

**Figure 11:**
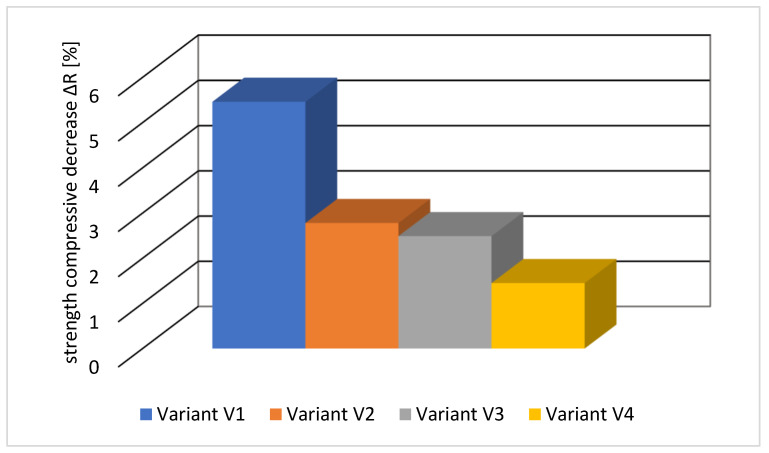
The average decrease in strength of tested concrete samples.

In all tested variants, no loss of mass for samples subjected to freezing and thawing cycles was recorded. The results of the strength decrease tests are shown in Figure 11.

The analysis of the freeze-thaw tests showed that the assumed F150 class was obtained for all four concrete mix recipes. None of the tested samples was cracked or scratched. Concrete, according to variant V1, shows the greatest decrease in compressive strength, i.e., 5.46%. The smallest decrease in strength, i.e., 1.45%, was observed for concrete according to variant V4. The reason for the better results in the case of variant V4 is a more airtight structure compared to other concretes due to the use of both a sealing admixture and fly ash, which reduces the access of water to the interior of the concrete.

## 4. Conclusions

Waterproof concrete is a modern building material that allows for replacing traditional solutions using external insulation in the form of coatings or membranes. Appropriate design of the concrete mix and execution of the concrete ensures the extension of the service life of the concrete structures. Based on the obtained results of the conducted tests, it can be stated that:

1. The applied sealing admixture had a positive effect on the tightness of the concrete and thus improved the frost resistance of the concrete. A 56% decrease in water penetration from 41 mm for the V1 series to 18 mm for the V2 series was observed.
2. Fly ash enhanced the effect of the sealing admixture, additionally limiting the depth of water penetration under pressure. For the V4 series, the depth of water penetration was 16 mm, which is a reduction in this parameter by 61%.
3. The addition of fly ash to the concrete mix improved its workability and increased the air content in the concrete mix. It reduced the compressive strength in the first stage of maturation but increased it later, i.e., after 90 days.
4. The sealing admixture has a positive effect on the tightness of the concrete, which is reflected in the compressive strength. After 90 days of curing, it was found that in concrete without fly ash, the strength increased by 1.1% (series V1 and V2), while in the presence of fly ash by 1.2% (series V2 and V3).
5. Analysing the properties of the tested concretes, it can be concluded that the most favorable results were obtained for the V4 series, which contained the addition of fly ash and a sealing admixture.

In the absence of detailed guidelines for the design of watertight concretes the test results can be used by technologists and concrete designers as guidelines in the selection of the composition of watertight concrete.

Future research will include an analysis of the possibility of using fly ash from municipal waste incineration in the composition of watertight concrete.

## Figures and Tables

**Table 1:** Chemical composition of cement CEM II/B-S 42.5N NA.

Chemicals	Formula	Content [wt.%]
Calcium oxide	CaO	53.37
Silicon dioxide	SiO_2_	26.73
Aluminium dioxide	Al_2_O_3_	7.06
Iron oxide	Fe_2_O_3_	2.81
Magnesium oxide	MgO	3.51
Sulfur oxide	SO_3_	2.09
Chlorine	Cl	0.059
Sodium oxide	Na_2_O_eq_	0.66
insoluble parts		3.49
roasting losses		1.77

**Table 2:** Mechanical and physical properties of cement CEM II/B-S 42,5N NA.

Curing Time [day]	Compressive Strength [MPa]		Physical Properties of Cement		
	Average	Required			
2	21.6	≥10.0	specific surface [cm^2^/g]	Soundness (expansion) [mm]	Initial setting time [min]
28	58.7	≥42.5 ≤62.5	4091.0	0.6	221.0

**Table 3:** Chemical composition of fly ash.

Chemicals	Formula	Content [wt.%]
Silicon dioxide	SiO_2_	52.2
Aluminium dioxide	Al_2_O_3_	28.4
Iron oxide	Fe_2_O_3_	8.1
Calcium oxide	CaO	4.1
Magnesium oxide	MgO	2.4
Sulfur trioxide	SO_3_	0.4
Sodium oxide	Na_2_O	0.9
Potassium oxide	K_2_O	2.7
Chlorine	Cl	0.008
Loss of ignition	LOI	2.6

**Table 4:** Recipes of concrete mixes per 1 m^3^ of concrete.

No	Component of the Concrete Mix	Density (g/cm^3^)	Mass [kg]			
			Variant of Concrete Mix			
			V1	V2	V3	V4
1.	Sand 0/2 mm	2.62	700.00	700.00	682.00	682.00
2.	Gravel 2/8 mm	2.58	501.00	501.00	488.00	488.00
3.	Gravel 8/16 mm	2.58	619.00	619.00	603.00	603.00
4.	Cement CEM II/B-S 42.5N NA	3.05	360.00	360.00	360.00	360.00
5.	Fly ash	2.20	0.00	0.00	40.00	40.00
6.	Water	1.00	156.00	153.00	156.00	153.00
7.	Plasticizer (BV3M)	1.19	1.80	1.80	1.70	1.70
8.	Superplasticizer (Visco Crete 5)	1.07	3.60	3.60	3.60	3.60
9.	Sealing admixture (WT-200P)	1.10	0.00	3.60	0.00	3.60

**Table 5:** Results of consistency of concrete mix made with slump test.

Time after Which the Test Is Performed [min]	Concrete Mix Cone Slump [mm]			
	V1	V2	V3	V4
5	50	60	50	60
15	80	85	85	90
30	95	95	95	100
45	90	90	90	90
60	90	90	90	90

**Table 6:** Air content in the concrete mix.

Air Content in Concrete Mix [%]			
Variant			
V1	V2	V3	V4
2.3	2.2	2.6	2.4

**Table 7:** Compressive strength.

Variant	Average Compressive Strength [MPa]				
	Cubic Samples with Side of 100 mm				
	2 Days	7 Days	28 Days	56 Days	90 Days
V1	28.0	48.4/↑72.9%	69.9/↑44.4%	72.6/↑3.9%	72.7/↑0.1%
SD *	1.49	1.15	1.55	1.54	1.86
V2	26.5	47.0/↑77.4%	68.5/↑45.7%	73.3/↑7.0%	73.5/↑0.3%
SD *	1.09	0.88	1.62	1.03	1.91
V3	25.6	47.6/↑85.9%	69.1/↑45.2%	72.1/↑4.3%	73.8/↑2.4%
SD *	0.99	0.99	1.40	1.42	1.12
V4	24.5	45.6/↑86.1%	70.7/↑55.0%	71.7/↑1.4%	74.7/↑4.2%
SD *	1.11	0.86	1.51	1.42	1.76
	**Cubic Samples with Side of 150 mm**				
	**2 Days**	**7 Days**	**28 Days**	**56 Days**	**90 Days**
V1	-	48.6	68.6/↑41.0%	-	70.9/↑3.9%
SD *		1.22	1.48		1.38
V2	-	52.0	68.3/↑31.4%	-	71.4/↑4.5%
SD *		1.65	1.58		1.84
V3	-	46.2	69.6/↑50.7%	-	73.8/↑6.0%
SD *		1.35	1.22		1.22
V4	-	48.5	71.0/↑46.4%	-	75.2/↑5.9%
SD *		1.37	1.49		1.57

*—Standard deviation; ↑increase in compressive strength [%] is shown in green

## Data Availability

The data presented in this study are available on request from the corresponding author.
